# Public preferences for engagement in Health Technology Assessment decision-making: protocol of a mixed methods study

**DOI:** 10.1186/s12911-015-0176-0

**Published:** 2015-07-14

**Authors:** Sally Wortley, Allison Tong, Emily Lancsar, Glenn Salkeld, Kirsten Howard

**Affiliations:** Sydney School of Public Health, The University of Sydney, Camperdown, 2006 Australia; Centre for Kidney Research The Children’s Hospital, Westmead Corner Hawkesbury and Hainsworth Street, Westmead, 2145 Australia; Centre for Health Economics, Monash University, Clayton, 3800 Australia

**Keywords:** Health technology assessment, Discrete choice study, Decision-making, Mixed methods, Public engagement

## Abstract

**Background:**

Much attention in recent years has been given to the topic of public engagement in health technology assessment (HTA) decision-making. HTA organizations spend substantial resources and time on undertaking public engagement, and numerous studies have examined challenges and barriers to engagement in the decision-making process however uncertainty remains as to optimal methods to incorporate the views of the public in HTA decision-making. Little research has been done to ascertain whether current engagement processes align with public preferences and to what extent their desire for engagement is dependent on the question being asked by decision-makers or the characteristics of the decision. This study will examine public preferences for engagement in Australian HTA decision-making using an exploratory mixed methods design.

**Methods/Design:**

The aims of this study are to: 1) identify characteristics about HTA decisions that are important to the public in determining whether public engagement should be undertaken on a particular topic, 2) determine which decision characteristics influence public preferences for the extent, or type of public engagement, and 3) describe reasons underpinning these preferences. Focus group participants from the general community, aged 18–70 years, will be purposively sampled from the Australian population to ensure a wide range of demographic groups. Each focus group will include a general discussion on public engagement as well as a ranking exercise using a modified nominal group technique (NGT). The NGT will inform the design of a discrete choice study to quantitatively assess public preferences for engagement in HTA decision-making.

**Discussion:**

The proposed research seeks to investigate under what circumstances and how the public would like their views and preferences to be considered in health technology assessments. HTA organizations regularly make decisions about when and how public engagement should occur but without consideration of the public’s preferences on the method and extent of engagement. This information has the potential to assist decision-makers in tailoring engagement approaches, and may be particularly useful in decisions with potential for conflict where clarification of public values and preferences could strengthen the decision-making process.

## Background

There is increasing recognition of the role and importance of public engagement in health technology assessment (HTA) decision-making. HTA is a multidisciplinary process that summarises information about the medical, social, economic and ethical issues related to the use of a health technology. It aims to inform the formulation of safe, effective, health decisions and policies that are patient-focused and represent value for money [1]. Public engagement is a means to achieve these aims, because it increases the transparency of the process and provides a mechanism to consider social values related to the technology being assessed [2–4]. Most organizations involved in HTA conduct some form of consultative or participative public engagement to inform decision-making [5]. However, HTA organizations (HTAOs) have traditionally made their own independent judgments regarding who to engage, the method of engagement and at what point in the HTA continuum to engage. The basis on which these decisions are made is unclear, as there is a lack of published evidence as to when public engagement should be undertaken, how it should be obtained and under what circumstances it is most effective [6].

Generally public engagement is used as a broad term to cover a number of different types of dialogue; from basic information provision to more complex deliberation and collaboration. Many frameworks have been published to conceptualize the differences between types of engagement with most conceptualizing a continuum whereby each successive type represents an increasing degree of influence, commitment and/or participation [7, 8]. In HTA, public engagement methods can include basic information provision, to consultative mechanisms such as focus groups and consumer representation on decision making committees [9], to more complex methods of deliberation including citizens juries and consensus panels [5].

Public engagement frameworks suggest that the complexity and the impact of a topic (in this case the HTA) should influence the type of engagement undertaken by a decision-making organization [10]. The difficulty arises in defining what is meant by ‘complexity’ and ‘impact’ in HTA. Some authors have suggested that complexity relates to the nature of the evidence base: this includes issues such as the type of the technology under review, the research question, and/or the quantity and quality of the evidence available [11].

In contrast, ‘impact’ relates to the inferred effect of a decision on the broader population [12] and is often portrayed as relating to the interests of the stakeholders involved [11]. In HTA these stakeholders include health professionals, pharmaceutical companies, device manufacturers as well as the public and patient organizations. The interests of these groups are driven by both the characteristics of the target population and the disease (condition) as well as the technology. As such, understanding the effect of a decision involves issues such as the number of people with the condition, their characteristics (e.g. age, ethnicity), the perceived benefit of the technology (relative to the severity/burden of the condition), the availability of other treatment options, whether expert opinion is divided on the effectiveness of the technology and the cost of the technology [13].

Issues around complexity and impact have been described in the literature as external decision-making context factors [12], i.e., those factors that are fixed, uncontrollable and cannot be manipulated by decision-makers but to do with the context of the HTA itself. (such as prevalence of the condition, quality of the evidence). Internal decision-making context factors, on the other hand, are factors relating to the decision-making process [12]. Such factors include the time and resources available to the HTAO, the organizational cultural of the HTAO. These factors are rarely made explicit in public engagement frameworks, but have been noted as being just as influential in determining when and how members of the public would be engaged [11, 14, 15].

Although a number of studies [16, 17] have been undertaken in the public engagement and HTA sphere, most have examined the importance of these factors one at a time. Additionally many of these studies are from the perspective of the decision maker and none have specifically considered the views of the general public. It may be that in some circumstances the public are happy not to be involved in decision-making [18, 19]; particularly if the existing systems are transparent and already capture a diversity of views [20]. Understanding the relative value of such factors (or what we refer to as ‘characteristics of a HTA decision’) depends upon considering several of them simultaneously and allowing individuals to weigh and trade-off these factors to clarify which aspects might be relevant under what circumstances [21]. This will enable us to determine for which policy questions the public consider public engagement would be best undertaken. The next step would be for policy makers to clarify what they want from the public in these circumstances [20] including the extent to which they are willing to cede or not, to the public’s views.

The objectives of this study are to determine if and how the public would like their views and preferences to be considered in HTAs and whether this differs depending upon the question posed by decision-makers and/or the characteristics of the HTA.

## Methods/Design

**Fig. 1:**
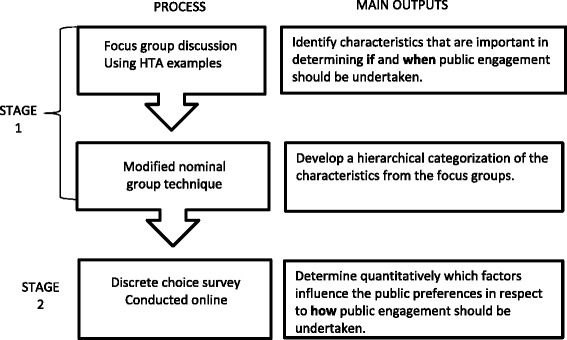
Sequence of the public preferences for engagement in Health Technology Assessment decision‐making mixed methods study

In order to determine the public’s preferences on engagement in HTA, we will undertake a mixed methods study involving focus groups and a discrete choice experiment (DCE). The approach will involve collecting both qualitative and quantitative data from focus groups, followed by an online DCE to quantitatively assess the public’s preferences for engagement in HTA decisions and the trade-offs individuals are willing to make between engagement methods depending on the characteristics of the individual HTA decision. Fig. 1 outlines the sequence for the proposed study. Stage one will focus on views of the public in respect to public engagement and HTA decision-making whereas stage two will explore the link between these views and different engagement methods. As there is an extensive body literature on public engagement [22], a series of literature reviews will be undertaken to inform both stages. These will be used to outline what is already known on the topic and to better define the tools and to assist with interpretation of findings.

Focus groups and nominal group technique

Focus group discussions incorporating a modified nominal group technique will be used, alongside a review of the literature, to establish the attributes for the DCE. Nominal group techniques (NGT) are a formal consensus development method based on structured group discussion [23]. This method supports idea generation and enables exploration and debate of views within a group; which is particularly useful in situations where participants are likely to have diverse views on a subject or where limited existing research evidence is available [24]. This technique also prevents individual participants from controlling the discussion, and allows all participants the opportunity to share their suggestions and opinions [25]. In comparison to traditional NGT, generation of ideas in this study will be undertaken as part of the focus group rather than as an individual exercise. Such techniques (both focus groups and modified NGT) have been used successfully in conjunction with DCEs [24, 26, 27].

Discrete choice experiments

DCEs are quantitative surveys in which respondents are asked to choose between hypothetical alternatives defined by a set of attributes with varying levels. The method is underpinned by the theory that goods and services, including health care services or policies, can be described in terms of separate attributes [28, 29]. The levels of attributes are varied systematically in a series of questions and respondents choose the option that they prefer for each question. People choose their most preferred option, interpreted as the option from which they derive the highest ‘value’ or ‘utility’ [30]. From these choices, a mathematical function is estimated in a random utility framework which estimates the probability with which options are chosen; subsequently the relative importance or value of different attributes can be investigated. Other data collected in the survey, including attitudinal questions and sociodemographic information, can also be included as explanatory variables. Therefore, DCEs can explore which attributes are driving public preferences, the trade-offs between attributes that people are willing to accept, and how changes in attributes can lead to changes in preferences.

Attribute identification and selection is an important part of DCE design. The process of attribute identification determines the options that the individual sees and ultimately shapes the policy conclusions that can be drawn from the DCE [31]. Good practice in DCE design is for qualitative work to drive the identification and selection of attributes. This allows researchers to explore and understand concepts that will ultimately guide the design, development and analysis of the DCE [30] and assist in increasing the validity of the design and the analysis [32]. Therefore, we will use a combination of qualitative and quantitative methods and the existing HTA public engagement literature, to determine which attributes will be included in our DCE [26]. Such attributes may cover both issues of ‘complexity’ and ‘impact’ of a topic including severity of the condition, number of people with the condition who could benefit, certainty of the evidence and availability of alternatives [21, 33]. This literature will also inform the conduct of our focus groups.

### Study design

#### Stage one: Focus/nominal groups

The focus group discussions will centre on participants’ views on public engagement in Australian HTA decision-making. All focus/nominal groups will be begin with a brief introduction to HTA. Following this each focus/nominal group discussion will then have four main phases: discussion around public engagement practices in HTA and the adequacy of these methods; preliminary questions about the characteristics of a HTA that are important in decision-making, a group discussion of the characteristics (or factors) affecting decision-making and public engagement, and an individual ranking exercise involving the factors identified from the group discussion [34]. Examples of recent HTA decisions will be used to prompt discussion.

The aims of the focus/nominal group component are to:

1. explore the views of the public in respect to public engagement in HTA decision-making,
2. identify characteristics (factors) about individual HTAs (such as seriousness of the problem, number of people affected, quality of the evidence) that are important in determining whether public engagement should be undertaken on a particular topic,
3. develop a rank ordering of the factors elicited in aim 2 to be used in a DCE on public engagement in HTA, and
4. describe reasons underpinning their choices and rankings.

A minimum of six groups with approximately 10 participants per group will be convened. All groups will be conducted in metropolitan areas of NSW and run for around 2 hours. Participants will be recruited via a market research company and will be grouped by age to facilitate communication with their peers and because age is known to be a factor influencing participation preferences in health decision making [35, 36]. Purposive sampling will be undertaken to ensure a balance of numbers between male and female participants, cultural backgrounds, education and employment history, socioeconomic and parental status [37]. Prior to each group discussion participants will receive a participant information sheet about the study, and will be asked to sign a consent form before each focus groups begins. Each participant will receive AU $100 at the end of the focus group to cover time and travel costs.

#### Selection of HTA examples (case studies)

Four case studies of recent HTA decisions will be presented as part of the focus groups:

1. Ipilimumab for advanced stage melanoma
2. injection of Botulinum toxin for chronic migraine
3. magnetic resonance imaging (MRI) for diagnosis of Crohn’s disease
4. hypobaric oxygen therapy (HBOT) for chronic skin ulcers.

The details presented in these case studies will be based on publically available data from recent Australian HTA reports reviewed by either the Medical Service Advisory Committee [38, 39] or Pharmaceutical Benefits Advisory Committee [40, 41]. It is not intended that these examples be the focal point of the groups, rather that they offer concrete examples to prompt discussion. The four case studies reflect HTAs of potentially varying ‘impact ‘and ‘complexity’ and thus are intended to prompt different responses from the participants in regards to public engagement. Additionally, the examples vary in terms of the public engagement processes actually employed. It is anticipated that this will assist in elucidating issues around the use of different methods of public engagement in HTA. For example if the public considers complex decisions and those of potentially greater impact warrant public engagement, then it should follow that the characteristics reflected in the advanced melanoma and chronic migraine case studies will be raised in the focus group. This is because both these examples require trade-offs between different social values (e.g., seriousness of the problem, uncertain benefit, costs). In contrast, the characteristics of the Crohn’s case study, in terms of both complexity and impact, are considered to be more straightforward and likely to require fewer trade-offs.

#### Focus group questions

Focus/nominal group participants will be asked a range of open- and closed-ended questions, including broad questions on public engagement and HTA as well as more specific questions on the case studies. These questions will assist in the generation of a list that can be used in the modified nominal group exercise. From the list of decision aspects important in determining if public engagement should be undertaken, participants will be asked to rank the factors from 1 (most important) to 10 (least important).

### Data collection and analysis

#### Qualitative

Focus group discussions will be audio-recorded and transcribed. Transcripts will be imported into the computer software package Nvivo 10 which will be used to facilitate coding and analysis of the qualitative data. Concepts relating to HTA and public engagement will be identified inductively from the data. Similar concepts will be grouped into themes [42]. We will also identify relationships among themes. The analysis will be performed for all groups collectively; we will also explore whether any differences exist across sub-groups. The preliminary analysis will be discussed among all investigators to ensure that the full breadth and depth of data are captured in the results.

#### Quantitative

Individual respondent rankings will be used to calculate importance scores for each factor (or characteristic) identified in the focus groups. The highest-ranked factor for each respondent will be given 10 points, the next most important given 9, and so on, progressively down to least important being given 1. Factors not included in an individual’s top 10 will be given a score of 0. The individual rankings will be summed to derive rank orders at the group and overall levels; mean and median weighting importance scores will also be calculated, as well as the percentage of all respondents who ranked each factor in their top ten. Other methods of analysis of the group data such as the frequency and proportion of participants who included a factor in their ranking list will also be undertaken [43].

#### Stage two: Discrete choice experiment (DCE)

The thematic analysis, in conjunction with the strength and frequency of each factor within a theme, will be used to determine the key attributes [44]. Once the attributes and levels have been decided based on the qualitative work and the literature, a statistically efficient DCE design will be generated [45]. It is likely attributes will include, but not be limited to, factors such as prevalence of the condition, cost to the taxpayer, size of health gain. Once an initial design has been created, it will be piloted in a sample of 50 participants and preliminary models estimated. Parameter estimates from these will be used to generate the final efficient design for the main discrete choice study. In addition to the discrete choice questions, information on socio-demographic characteristics and exposure to health decision-making processes of respondents will also be collected.

#### Aims of the DCE component

1. to assess if the public would like their preferences to be considered in HTA decision-making
2. to determine how the public would like their views and preferences to be considered in HTA decision-making
3. to determine quantitatively which factors influence the public’s preferences in respect to how public engagement should be undertaken for individual technology decisions.

#### Data collection and analysis

The DCE survey will be conducted using a web-based questionnaire administered to a respondent sample broadly representative of the Australian public, recruited using quota sampling based on age and gender. Respondents will be recruited by a market research company with an existing online panel and experience in administering online choice-based surveys. The final sample size required will be based upon the characteristics of the design itself, such as the number of attributes included, the attribute level range, the number of choice scenarios presented, the number of alternatives in each choice set and the size and direction of prior parameters obtained from the pilot study. The DCE will also include an opt-out option as well as scenario that describe the status quo in relation to public engagement. To ensure that we are able to explore interactions between attributes and between attributes and socio-demographic factors, along with unobserved heterogeneity we anticipate a sample size of at least 1000 respondents.

The results from this survey will inform policy by highlighting the factors that influence community preferences for engagement with HTA decision making. We will initially use a mixed multinomial logit (MMNL) (also known as random parameters logit, RPL) model using a panel specification for the analysis. A panel specification of the model allows for non-independence of observations provided by the same respondent; that is it can account for correlations amongst the multiple choices made by the same individual. We will also explore the use of alternative model specifications such as panel latent class models and generalized multinomial logit models to estimate preferences for community engagement. We will examine interactions between attributes, and between attributes and population characteristics (for example, age, gender, income, education, non-english speaking background) before deciding on a final model specification. Model results will expressed as parameter estimates (β), the odds of choosing one option instead of another (and 95 % confidence intervals of the odds ratios) and *p*-values. Acceptable trade-offs between attributes will also be calculated.

### Ethical considerations

This study has been approved by the University of Sydney Ethics Committee (2014/053 and 2014/1022). Confidentiality and anonymity of the data will be strictly maintained. Digital recording of the focus groups will only take place after written informed consent is obtained from participants. Participants will not be identifiable in any transcripts, or in any publications. It will be made clear to all participants that they have the right to withdraw from the research at any point in time.

## Discussion

This study will use qualitative and quantitative methods to determine the Australian public’s preferences for engagement in HTA. It will build upon other studies of public engagement and social values in HTA, but will focus on ordinary or lay citizens rather than stakeholder groups such as patients or decision-makers. Stage one of the study will generate two separate outputs: a qualitative analysis of issues around public engagement, prioritization and resource allocation in health technology, and a ranked list of the characteristics that the public considers the most important in determining whether or not public engagement should be undertaken in HTA. Stage two of the study (i.e., the DCE) will allow quantification of the importance of each attribute (characteristic) in determining how public engagement should be undertaken in Australian HTA. Our research will produce a uniquely comprehensive understanding of the preferences of the public on when and how they would like engagement; this information can be used by health care agencies and decision-makers to tailor public engagement methods and inform future research on public engagement methodology.

## References

[1] Sansom L (2013). HTA and Value: a commentary. Int J Technol Assess Health Care.

[2] Littlejohns P, Weale A, Chalkidou K, Faden R, Teerawattananon Y (2012). Social values and health policy: a new international research programme. J Health Organ Manag.

[3] Facey K, Boivin A, Gracia J, Hansen HP, Lo Scalzo A, Mossman J (2010). Patients’ perspectives in health technology assessment: a route to robust evidence and fair deliberation. Int J Technol Assess Health Care.

[4] Nilsen ES, Myrhaug HT, Johansen M, Oliver S, Oxman AD (2006). Methods of consumer involvement in developing healthcare policy and research, clinical practice guidelines and patient information material. Cochrane Database Syst Rev.

[5] Whitty JA (2013). An international survey of the public engagement practices of health technology assessment organizations. Value Health.

[6] Mitton C, Smith N, Peacock S, Evoy B, Abelson J (2009). Public participation in health care priority setting: a scoping review. Health Policy.

[7] Arnstein S (2011). A ladder of citizen participation. LeGates, Richard T; Stout, Frederic Frederic, Stout (Hrsg).

[8] Rowe G, Frewer LJ (2005). A typology of public engagement mechanisms. Sci Technol Hum Values.

[9] Menon D, Stafinski T (2011). Role of patient and public participation in health technology assessment and coverage decisions. Expert Rev Pharmacoecon Outcomes Res.

[10] Nabatchi T. Putting the 'public' back in public values research: designing participation to identify and respond to values. Public Adm Rev. 2012;72:699–708.

[11] Gauvin FP, Abelson J, Giacomini M, Eyles J, Lavis JN (2010). It all depends: Conceptualizing public involvement in the context of health technology assessment agencies. Soc Sci Med.

[12] Dobrow MJ, Goel V, Upshur REG (2004). Evidence-based health policy: context and utilisation. Soc Sci Med.

[13] Stafinski T, Menon D, McCabe C, Philippon DJ (2011). To fund or not to fund. Pharmacoeconomics.

[14] Rychetnik L, Carter SM, Abelson J, Thornton H, Barratt A, Entwistle VA (2013). Enhancing citizen engagement in cancer screening through deliberative democracy. J Natl Cancer Inst.

[15] Watt AM, Hiller JE, Braunack-Mayer AJ, Moss JR, Buchan H, Wale J, et al. The ASTUTE Health study protocol: Deliberative stakeholder engagements to inform implementation approaches to healthcare disinvestment. Implement Sci. 2012;7:101. 10.1186/1748-5908-7-101 PMC3520863 23088222

[16] Street JM, Braunack-Mayer AJ, Facey K, Ashcroft RE, Hiller JE. Virtual community consultation? Using the literature and weblogs to link community perspectives and health technology assessment. Health Expect. 2008;11:189–200. 10.1111/j.1369-7625.2007.00484.x PMC5060433 18430153

[17] Messina MJ, Grainger DL (2012). A pilot study to identify areas for further improvements in patient and public involvement in health technology assessments for medicines. The Patient: Patient-Centered Outcomes Research.

[18] Lomas J (1997). Reluctant rationers: public input to health care priorities. J Health Serv Res Policy.

[19] Litva A, Coast J, Donovan J, Eyles J, Shepherd M, Tacchi J (2002). The public is too subjective: public involvement at different levels of health-care decision making. Soc Sci Med.

[20] Lehoux P, Daudelin G, Demers-Payette O, Boivin A (2009). Fostering deliberations about health innovation: What do we want to know from publics?. Soc Sci Med.

[21] Stafinski T, Menon D, Marshall D, Caulfield T (2011). Societal Values in the Allocation of Healthcare Resources. The Patient: Patient-Centered Outcomes Research.

[22] Sarrami-Foroushani P, Travaglia J, Debono D, Braithwaite J (2014). Key concepts in consumer and community engagement: a scoping meta-review. BMC Health Serv Res.

[23] Van de Ven AH, Delbecq AL (1972). The nominal group as a research instrument for exploratory health studies. Am J Public Health.

[24] Howard K, Jan S, Rose J, Chadban S, Allen RD, Irving M (2011). Community Preferences for the Allocation & Donation of Organs-The PAraDOx Study. BMC Public Health.

[25] Cantrill JA, Sibbald B, Buetow S (1996). The Delphi and nominal group techniques in health services research. Int J Pharm Pract.

[26] Hiligsmann M, van Durme C, Geusens P, Dellaert BG, Dirksen CD, van der Weijden T (2012). Nominal group technique to select attributes for discrete choice experiments: an example for drug treatment choice in osteoporosis. Patient Prefer Adherence.

[27] Scuffham PA, Ratcliffe J, Kendall E, Burton P, Wilson A, Chalkidou K (2014). Engaging the public in healthcare decision-making: quantifying preferences for healthcare through citizens juries. BMJ open.

[28] Lancaster KJ (1966). A new approach to consumer theory. J Polit Econ.

[29] Ryan M, Kolstad J, Rockers P, Dolea C (2012). How to conduct a discrete choice experiment for health workforce recruitment and retention in remote and rural areas: a user guide with case studies.

[30] Lancsar E, Louviere J (2008). Conducting discrete choice experiments to inform healthcare decision making. Pharmacoeconomics.

[31] Coast J, Al-Janabi H, Sutton EJ, Horrocks SA, Vosper AJ, Swancutt DR, et al. Using qualitative methods for attribute development for discrete choice experiments: issues and recommendations. Health Econ. 2012;21:730–41. 10.1002/hec.1739 21557381

[32] Lancsar E, Swait J (2014). Reconceptualising the External Validity of Discrete Choice Experiments. Pharmacoeconomics.

[33] Guindo LA, Wagner M, Baltussen R, Rindress D, van Til J, Kind P (2012). From efficacy to equity: Literature review of decision criteria for resource allocation and healthcare decisionmaking. Cost Eff Resour Alloc.

[34] Corner J, Wright D, Hopkinson J, Gunaratnam Y, McDonald JW, Foster C (2007). The research priorities of patients attending UK cancer treatment centres: findings from a modified nominal group study. Br J Cancer.

[35] Thompson SC, Pitts JS, Schwankovsky L (1993). Preferences for involvement in medical decision-making: situational and demographic influences. Patient Educ Couns.

[36] Say R, Murtagh M, Thomson R (2006). Patients preference for involvement in medical decision making: a narrative review. Patient Educ Couns.

[37] Teddlie C, Yu F (2007). Mixed methods sampling a typology with examples. J Mix Methods Res.

[38] Gordon L, Comans T, Scuffham PA. MRI for small bowel Crohn’s disease and fistulising perianal Crohn’s disease*.* Canberra; 2013

[39] Hoggan B, Cronin P, Camerson A, Goodall S. Hyperbaric Oxygen Therapy (HBOT) for the Treatment of Chronic Non-Diabetic Wounds and Non-Neurological Soft Tissue Radiation Injuries*.* Canberra; 2011

[40] PBAC. Ipilimumab, concentrate solution http://www.pbs.gov.au/info/industry/listing/elements/pbac-meetings/psd/2012-11/ipilimumab. Accessed 12th May 2014,

[41] PBAC .Botulinum Toxin Type A, injection http://www.pbs.gov.au/info/industry/listing/elements/pbac-meetings/psd/2013-07/botulinum. Accessed 12th May 2014

[42] Braun V, Clarke V (2006). Using thematic analysis in psychology. Qual Res Psychol.

[43] McMillan SS, Kelly F, Sav A, Kendall E, King MA, Whitty JA (2014). Using the Nominal Group Technique: how to analyse across multiple groups. Health Serv Outcomes Res Methodol.

[44] McMillan SS, Kelly F, Sav A, Kendall E, King MA, Whitty JA (2014). Using the Nominal Group Technique: how to analyse across multiple groups. Health Serv Outcomes Res Methodol.

[45] Rose JM, Bliemer MC (2009). Constructing efficient stated choice experimental designs. Transp Rev.

